# Social frame and tax compliance modulate electrophysiological and autonomic responses following tax-related decisions

**DOI:** 10.1038/s41598-019-41156-7

**Published:** 2019-03-18

**Authors:** Michela Balconi, Davide Crivelli, Cinzia Castiglioni, Edoardo Lozza

**Affiliations:** 1 Research Unit in Affective and Social Neuroscience, Catholic University of the Sacred Heart, Milano, Italy; 2 Department of Psychology, Catholic University of the Sacred Heart, Milano, Italy

## Abstract

Given the intrinsic complexity of cognitive and affective processes affecting how people reason about taxes and their decisions to be compliant with such social duty, we aimed at exploring those latent processes by combining the analysis of their central and peripheral physiological correlates. We asked participants to make realistic economic decisions concerning tax-payment and manipulated the social vs. individual decisional frame. In addition, we took into account the potential role of tax-compliance trait. Thirty self-employed professionals took part in the study and completed a public good game while their autonomic (skin conductance – SC – and heart rate – HR) and neural brain (electroencephalography – EEG) activities were recorded. The analysis of physiological responses during the feedback phase – where participants could be presented or not with a fiscal audit – highlighted: (i) increased tonic SC levels and theta activity in the social condition than in the individual one; (ii) increased HR values when a fiscal audit did not take place, especially in participants who presented an enforced tax-compliance trait. Present findings support the idea that classic economic theories of tax behaviour developed under the assumption that taxpayers act as rational and individualist agents do not provide a comprehensive account for the decision-making process. They add to available evidence highlighting the contribution of psychological and social-affective variables to individuals’ decision-making processes to pay or evade taxes and to their appraisal of the consequences of such choice, as suggested by the ‘slippery slope’ framework.

## Introduction

Paying taxes is a key element of a well-functioning society, as the economic development of a country can be severely hampered by lower public revenues^1^. Nonetheless there is still limited understanding as to why people pay their taxes.

Classic economic models of tax compliance^2,3^ assume that taxpayers behave as rational agents and follow a cost-benefit analysis that takes into account the income loss if caught evading (penalty) and the probability of being caught (audited). Empirical research, however, has shown that such models fail to predict realistic tax evasion level, since a large fraction of people pay taxes despite the rather low levels of fines and probability of auditing^4^. This implies that tax compliance decisions must be influenced by motivations not fully captured by the basic economics-of-crime approach. Schmölders^5^ was one of the firsts that advocated the need to use psychology to understand fiscal behaviour, by introducing the construct of tax morale^6^. His work led to a different stream of research showing that taxpayers’ willingness to cooperate is influenced by a number of internal variables and psychological determinants, such as knowledge, values, attitudes, norms, tax morale and perception of tax authorities^7–11^. The complexity of the subject is also illustrated by the existence of different types of compliance, as suggested by the ‘slippery slope’ framework^12–14^. By integrating the economic assumptions of tax compliance (audits and fines) as well as the psychological and sociological determinants, two main forms of compliance can be identified: voluntary tax-compliance – i.e. a propensity that grounds on spontaneous willingness to cooperate following taxpayers’ attitude towards moral obligation to contribute to the public welfare – and enforced tax-compliance – i.e. a propensity that primarily grounds on taxpayers’ concerns of being audited and fined.

A further argument against believing that taxpayers are mere rational agents is the importance of social variables in the decision-making process. Paying taxes is a classic example of social dilemma, as it involves a tension between what is best for the individual and what is best for the society as a whole^15^. This tension is well documented in tax behaviour research. Despite their importance, individual psychological traits and personal norms cannot fully capture the complexity of the decision to be compliant. Social variables play a fundamental role too. The perceptions about the prevalence of tax cheating within one’s local community^16^, taxpayers’ attachment to their reference group or society^11^, general societal expectations as well as other taxpayers’ actual behaviour^17^ can all affect the level of tax compliance or tax evasion. To summarize, it appears that the decision to pay taxes is not merely based on an individual cost-benefit analysis – as classic economic models would suggest – but takes into account other psychosocial variables such as the social context in which the decision is made and one’s moral obligation towards the provision for the common good^18^.

Given the inherently hidden nature of tax evasion and the absence of direct observability of this phenomenon, data collection for studying tax compliance and its antecedents poses difficulties. In a recent review, Torgler^19^ outlined the major tools and exploratory methods used in the field of tax compliance research. Survey data have been widely used, especially to measure social norms or tax morale (e.g., World Value Survey, European Value Survey, German General Social Survey, etc.). The main limit of surveys, however, is that they mainly rely on self-report data. Laboratory experiments have been an essential tool of exploration in the tax compliance field too, although experiments to date have tended to rely mainly on behavioural data, with limited understanding of what happened during the experiment. Therefore, the compilation of neurobiological data during the experiments may allow a deeper understanding of such decision-making process. Nevertheless, only few examples can be found in the tax compliance literature.

In addition to being scant, the nature of neurobiological data collected for tax behaviour studies varies a lot. Functional magnetic resonance imaging (fMRI) evidence suggests that mandatory (tax-like) transfers to charity lead to neural activation in areas linked to reward processing^20^. At hormonal level, it has been found that high testosterone levels may inhibit tax evasion behaviour^21^. Skin conductance responses (SCR) evidence indicates that tax cheating is correlated with an emotional arousal before submitting the decision^22^, thus suggesting that tax evasion does not only result from the ‘cold’ and rational comparison between the monetary benefits and costs of evading taxes. Contrary to the findings of Coricelli *et al*.^22^, heart rate variability (HRV) data seem to indicate that higher psychic stress is related to an increase in tax compliance, rather than a decrease^23^. Finally, an experiment measuring EEG activity suggests that tax decision-making processes change accordingly to how tax authorities are perceived^24^.

In conclusion, the literature on tax compliance has shown the importance of both economic and psychological variables to explain taxpayers’ decisions to pay or evade taxes. Undoubtedly, tax compliance research may benefit from a deeper understanding of the biological foundations of individuals’ decision-making. Thus, the present study aims to explore such latent processes by integrating neurobiological data of different nature (i.e. EEG, SCL, SCR, and HR modulations). The study is also novel in that it investigates what happens during a feedback phase where participants are presented or not with a fiscal audit.

## Methods

### Sample

The sample was constituted by thirty self-employed professionals (13 women, 17 men; *range*_*age*_ = 25–65, *M*_*age*_ = 39.16, *SD*_*age*_ = 12.20). Real taxpayers are relevant in tax compliance research because students often lack real tax experience, and self-employed people are especially relevant because they have more opportunities to actually evade their taxes, since they pay taxes out-of-pocket^9^. All participants had normal or corrected-to-normal sight. None of them reported history of neurological or psychiatric disorders. They gave their written informed consent to participate in the study. The study and relative procedures followed the principles of the Declaration of Helsinki and were approved by the Ethics Committee of the Department of Psychology of the Catholic University of the Sacred Heart.

### Procedure

The experimental procedure included a computerized task devised to simulate decision-making processes concerning tax-payment, the recording of participants’ electroencephalographic and autonomic activity while they took such decisions, and the completion of a standardized questionnaire devised to explore tax compliance.

The experimental task was implemented in E-Prime2.0 software (Psychology Software Tools Inc., Sharpsburg, PA, USA) following the structure of a public goods game^25^ – i.e. an experimental economics task where players are asked to make economic decisions concerning whether and how much they would contribute for the creation of a shared public good. Going down to specifics, participants started with 100 € fixed income and the task included 40 rounds – i.e. 40 decisions. In each round participants were asked to pay the 20% of their taxable income. Again, while participants knew they could freely decide whether to pay or not to pay such tax, they also knew that they might receive fiscal audits and that, should they receive an audit and found non-compliant, they would have to pay a fine equal to 300% of the due amount – i.e. 60 €. Eight out of forty rounds included a fiscal audit. Participants were blinded with regard to when they would have received an audit. Each round then included a decision-making phase (duration: 2 seconds) and, after a brief 4-second blank, a feedback phase (duration: 4 seconds) where they were presented or not with a fiscal audit. The task also included two main experimental conditions. In the individual condition participants were told that they were the only agents who independently decided whether to pay the taxes. In the social condition participants were told that four other potential agents were deciding whether to pay the taxes in order to ensure public welfare, although they were blinded about others’ decisions. Such condition was included to properly investigate the potential effect of a social context on decision-making process, following previously described evidence. Rounds were equally divided between the two experimental conditions.

During the task, participants’ neural (EEG) and autonomic (skin conductance and heart rate) responses were continuously monitored and recorded for subsequent analysis (see “*EEG recording and reduction*” and “*Recordings and reduction of autonomic data*” sections).

In addition to the experimental task, participants’ subjective tax-compliance was measured via the Italian version of the Tax Compliance Inventory^26^, which allows classifying people according to two main forms of compliance: voluntary tax compliance vs. enforced tax compliance. The questionnaire is constituted by 10 statements. Participants were asked to express their degree of agreement with the statements on a 9-point Likert scale (1 = “I completely disagree” to 9 = “I completely agree”). Their responses were then used to classify them as voluntary taxpayers (N = 20) or enforced tax-payers (N = 10) according to their prevailing form of tax-compliance.

### EEG recording and reduction

EEG data were collected via a 16-channel V-Amp system (Brain Products GmbH, Gilching, Germany). The electrode montage included 15 standard electrode positions (10–20 International System; Jasper, 1958) – divided into frontal (F7, F3, Fz, F4, F8), central (C3, Cz, C4), temporal (T7 and T8), parietal (P3, Pz, P4) and occipital (O1, O2) sites – and earlobes references (Ag/AgCl electrodes). Data were sampled at 500 Hz, with a 0.01–250 Hz bandpass and a 50 Hz notch filter as input filters. Two additional electrodes were placed above and below of the left eye to keep track of ocular artifacts. Impedance of recording electrodes was monitored prior to data collection for each subject and was always below 5 kΩ.

EEG data were filtered offline by using a 0.5–50 Hz bandpass filter and then fed to an automated algorithm to detect and mark ocular artifacts for subsequent correction^27^. EEG responses relative to the feedback phase of each experimental round were then segmented and visually inspected for residual ocular, muscle, or movement artifacts (rejected epochs: 5%). EEG power spectra for artifact-free segments were finally computed via Fast Fourier Transform and averaged to calculate condition-specific power spectra. Finally, mean power values for primary EEG frequency bands (delta – 0.5–3.5 Hz, theta – 4–7.5 Hz, alpha – 8–12.5 Hz, and beta – 13–30 Hz) have been extracted from each condition-specific power spectra.

### Recordings and reduction of autonomic data

Autonomic data were collected via the integrated recording module of a Biofeedback2000^xpert^ system (Schuhfried GmbH, Mödling, Austria). The multipurpose sensor was placed in correspondence to the distal phalanx of the second finger of the non-dominant hand. Skin conductance (tonic and phasic activity, respectively skin conductance level - SCL and skin conductance response - SCR) and cardiovascular data (heart rate - HR) were sampled at 40 Hz and inspected for the presence of artifacts. Finally, autonomic activity collected during the feedback phase of each experimental round was segmented and averaged to calculate mean condition-specific SCL, SCR and HR modulations. Skin conductance data pertaining to four participants were excluded from subsequent statistical analyses due to the high amount of recording artifacts affecting the signals.

### Data analysis

The distribution of participants’ choices have been preliminarily explored via analysis of variance. Such preliminary mixed-design ANOVA model included Choice (Compliant vs. Non-compliant) and Condition (Individual vs. Social) as within-subject factors, and Tax-compliance (Voluntary vs. Enforced) as between-subject factor.

Behavioural data concerning participants’ choices to pay taxes have then been used to compute a positive response index, where the number of compliant choices (i.e. when participants accepted to pay taxes) have been weighted over the total amount of choices that they had expressed. A mixed-design ANOVA model has then been applied to such behavioural index, including Condition (Individual vs. Social) as within-subject factor and Tax-compliance (Voluntary vs. Enforced) as between-subject factor. To further explore potential associations between behavioural and physiological data, we also performed psycho-physiological correlation analyses. Namely, we computed Pearson correlation coefficients between the positive response index and physiological responses (EEG and autonomic data), taking into account experimental conditions (i.e. Individual vs. Social context, Audit vs. Non-audit) and participants’ form of tax-compliance (Voluntary vs. Enforced).

EEG power data and autonomic data concerning the feedback phase of the experimental task have been analysed via mixed-design ANOVA models. The models applied to autonomic data included Condition (Individual vs. Social) and Audit (Audit vs. Non-audit) as within-subject factors and Tax-compliance (Voluntary vs. Enforced) as between-subject factor. The models applied to EEG data included Area (Frontal, Central, Temporal-Parietal, Occipital), Condition (Individual vs. Social) and Audit (Audit vs. Non-audit) as within-subject factors and Tax-compliance (Voluntary vs. Enforced) as between-subject factor. Type-I Errors due to inhomogeneity of variances have been controlled by applying Greenhouse-Geisser correction to the degrees of freedom when needed. Pair-wise comparisons were used to further explore simple effects for statistically significant interactions. In order to account for multiple comparisons bias, Bonferroni correction to probability values have been applied while computing pair-wise comparisons. Partial eta squared was computed to assess the size of significant main and interaction effects.

## Results

### Behavioural data and psycho-physiological correlations

The preliminary analysis of participants’ choices highlighted a significant main effect of the factor Choice (*F[1*,2*8]* = 36.60, *p* < 0.001, *η*^2^_*p*_ = 0.567), with a greater amount of compliant choices than non-compliant ones (M_Comp_ = 14.08, SD_Comp_ = 4.55; M_N-comp_ = 4.37, SD_N-comp_ = 4.42). No other main or interaction effect was statistically significant.

**Figure 1:**
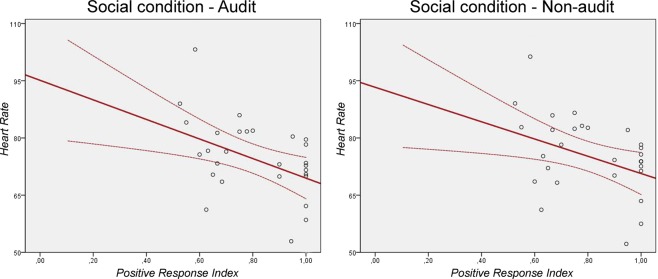
Significant correlations between the positive response index and heart rate values during both Audit (left panel) and Non-audit (right panel) trials of the Social condition. Solid lines represent global linear trends. Dashed lines represent 95% confidence intervals.

The ANOVA model applied to participants’ positive response indices did not highlight significant main or interaction effects. Conversely, psycho-physiological correlation analyses highlighted statistically significant inverse correlations between the positive response index and HR data only during the Social condition, regardless of the presence (*r* = −0.475, *p* = 0.008) or absence (*r* = −0.424, *p* = 0.019) of audit (see Fig. 1). Further exploratory analyses performed by splitting the sample based on participants’ tax-compliance highlighted that such inverse correlation between behavioural and HR data in the Social condition could be observed in both the Voluntary (Audit: *r* = −0.545, *p* = 0.013; Non-audit: *r* = −0.491, *p* = 0.028) and Enforced (Audit: *r* = −0.611, *p* = 0.061; Non-audit: *r* = −0.575, *p* = 0.082) subgroups, though for the Enforced subgroup correlation coefficients did not reach the statistical significance threshold.

### Autonomic data

**Figure 2:**
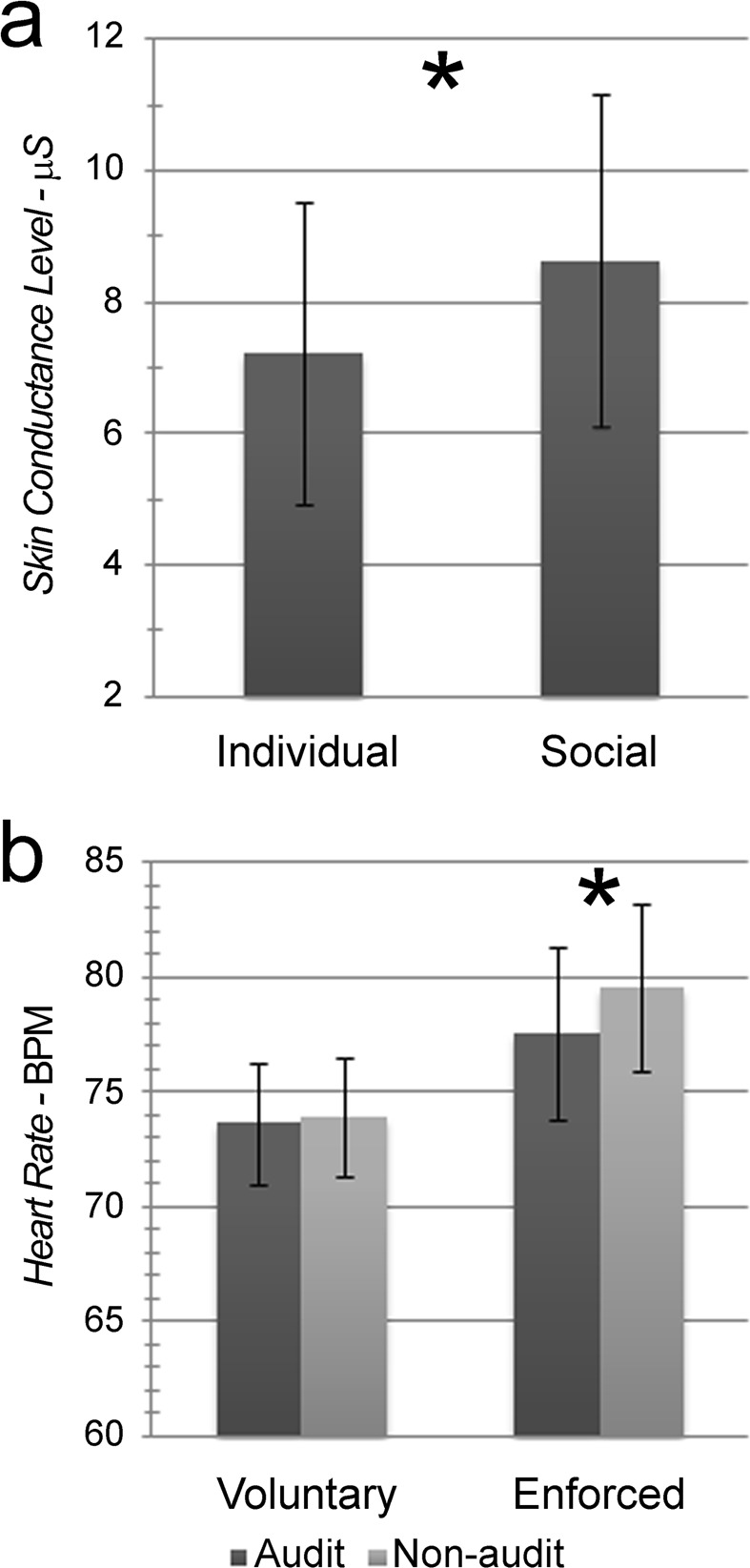
Autonomic activity during the feedback phase. Bars represent ± 1SE; the star marks statistically significant differences. (**a**) Significant modulation of Skin Conductance Level measures depending on the experimental condition: Individual *vs*. Social. (**b**) Modulations of Heart Rate measures depending on the tax-compliance style – Voluntary *vs*. Enforced – and on the presence *vs*. absence of a fiscal audit.

The analysis of SCL data highlighted a significant main effect of Condition (*F[1*,2*4]* = 4.33, *p* = 0.048, *η*^2^_*p*_ = 0.153, Fig. 2a), with higher skin conductance values in the Social than in the Individual condition (M_Soc_ = 8.64, SD_Soc_ = 2.53; M_Ind_ = 7.23, SD_Ind_ = 2.30). No other main or interaction effect was statistically significant.

The analysis of SCR data did not highlight significant main or interaction effects. The analysis of HR data highlighted a significant main effect of Audit (*F[1*,2*8]* = 8.25, *p* = 0.008, *η*^2^_*p*_ = 0.227) and a significant Tax-compliance X Audit interaction effect (*F[1,28]* = 4.60, *p* = 0.041, *η*^*2*^_*p*_ = 0.141; Fig. 2b). Globally, HR values were slightly higher in the Non-audit than in the Audit condition (M_N-aud_ = 76.74, SD_N-aud_ = 2.32; M_Aud_ = 75.63, SD_Aud_ = 2.30). Pair-wise comparisons further specified that increased HR values during Non-audit than Audit condition was specifically observed in the Enforced group (M_N-aud_ = 79.55, SD_N-aud_ = 3.64; M_Aud_ = 77.61, SD_Aud_ = 3.76; *p* = 0.005) and not in the Voluntary group (M_N-aud_ = 73.92, SD_N-aud_ = 2.58; M_Aud_ = 73.64, SD_Aud_ = 2.66; *p* > 0.05). No other main or interaction effect was statistically significant.

### EEG data

**Figure 3:**
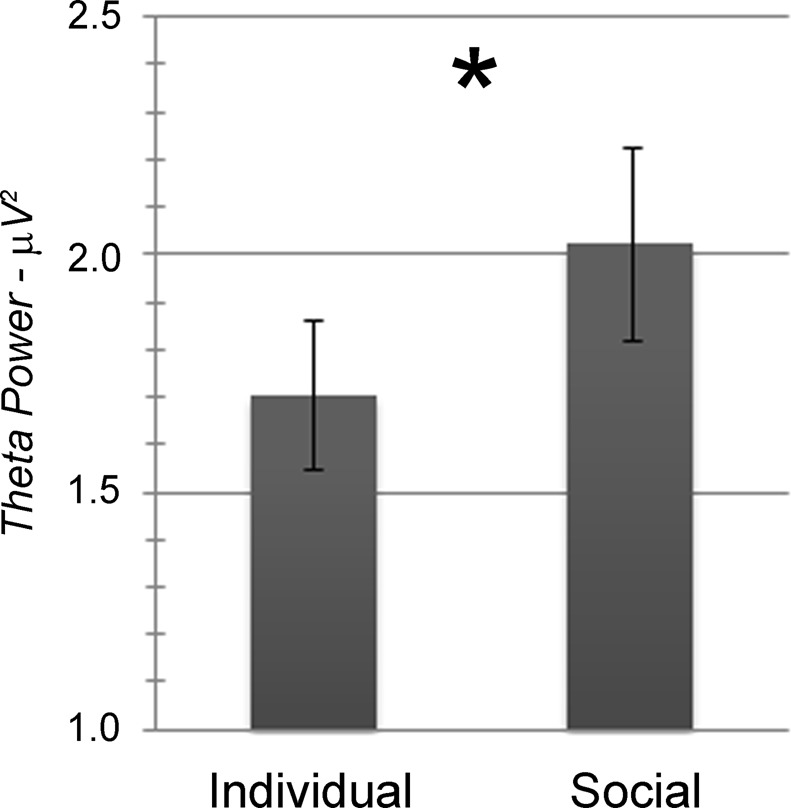
Significant modulation of EEG theta activity during the feedback phase depending on the experimental condition: Individual *vs*. Social. Bars represent ± 1SE; the star marks statistically significant differences.

The analysis of EEG power data concerning the theta frequency band highlighted a significant main effect of Area (*F[3,84]* = 53.40, *p* < 0.001, *η*^*2*^_*p*_ = 0.656) and a significant main effect of Condition (*F[1,28]* = 5.69, *p* = 0.024, *η*^*2*^_*p*_ = 0.169; Fig. 3). Theta activity was maximal at Frontal sites and gradually lower moving to Central, Temporal-parietal, and Occipital sites (M_Front_ = 3.28, SD_Front_ = 0.35; M_Centr_ = 1.77, SD_Centr_ = 0.16; M_Tem-par_ = 1.25, SD_Tem-par_ = 0.12; M_Occ_ = 1.15, SD_occ_ = 0.09; all *p* < 0.001 but for the Temporal-parietal vs. Occipital comparison, for which *p* > 0.05). Theta activity was also higher in the Social than in the Individual condition (M_Soc_ = 2.02, SD_Soc_ = 0.21; M_Ind_ = 1.71, SD_Ind_ = 0.16). No other main or interaction effect was statistically significant.

The analysis of EEG power data concerning the other standard frequency bands did not highlight significant main or interaction effects.

## Discussion

Given the intrinsic complexity of cognitive and affective processes affecting how people reason about taxes and their decisions to be compliant with such social duty, we aimed at exploring those latent processes by completing the exploration of behavioural choices with the analysis of their central and peripheral physiological correlates. To do so, we designed an ecological task and asked participants to make realistic economic decisions concerning whether and how much they would contribute for the creation of a shared public good, starting from a fixed income. During such simulation of the decision-making processes concerning tax-payment, EEG (frequency bands power) and autonomic (skin conductance – both tonic and phasic – and heart rate) data were collected. In particular, the present report focuses on a feedback phase – i.e. the moment when participants know they might receive a fiscal audit. The explorative analysis of covert measures of arousal and cognitive activity highlighted: (i) increased tonic skin conductance levels in the social condition, where they were told that four other potential agents were deciding whether to pay the taxes in order to ensure public welfare, than in the individual one; (ii) increased HR values during non-audit feedback phases with respect to when a fiscal audit took place, especially in participants who presented an enforced tax-compliance trait; (iii) increased theta EEG activity, maximal over frontal areas, during the social than the individual condition. Furthermore, the analysis of participants’ behavioural responses highlighted an overall preference for compliant choices, and additional psycho-physiological correlation analyses highlighted an inverse correlation between the amount of compliant responses and HR values, but only in the social condition.

A first main result is the consistent pattern of physiological activation presented by participants during the post-decisional phase in the social condition, regardless of whether they received or not a fiscal audit and regardless of their voluntary vs. enforced tax-compliance style. In the social condition participants took their decision about paying or not-paying taxes independently, as in the individual condition. Nonetheless, they were told that other agents were making the same economic decision, though they would not have been informed on whether they decided to pay taxes or be non-compliant. Therefore, the social dimension exerts, in this case, a framing effect. Such effect is simply elicited by reminding taxpayers of the virtual presence of other participants, without specifying neither others’ expectations (injunctive norm), nor their actual behaviour (descriptive norm). Namely, it affects participants’ post-decision processes by stressing the fact that they are not alone and that other people share their condition and are similarly asked to contribute to public welfare. The observed significant increase of physiological arousal and of specific components of the EEG frequency profile suggests that the introduction of such social dimension – even if just as a frame – might have had an effect on participants implicit attitudes toward the situation and, specifically, towards the consequences of their economic decisions. Rather than simply engaging in a utility calculation that compares the costs and benefits of their decision, as suggested by the presence of a sanctioning system (i.e., audits and fines), the social frame may have evoked an ethical decision frame^28^, where not only individual but also collective consequences are taken into account. Going down to specifics, the tonic component of electrodermal activity (i.e. SCL) is a robust and informative index of the activity of the sympathetic branch of the autonomous nervous system^29,30^, which regulates bodily activation and stress-related responses. Higher SCL values collected during the social condition with respect to the individual one may then suggest that a simple communication frame orienting participants’ attention to the presence of other social agents with similar responsibilities may heighten their level of vigilance and arousal. Further, it is worth noting that such physiological modulation was observed during the feedback phase, when participants had already decided whether to be compliant or not with tax-payment and could be presented or not with a fiscal audit. Given that they were actually in an examination phase, we suggest that arousal might have been modulated by the possibility of being exposed to social judgment, in line with available evidence on the effect of psychosocial factors on stress-related and arousal responses^31–33^.

Such interpretation is further supported by the complementary increase of theta EEG activity in the social condition, once again regardless of the presence or absence of a fiscal audit and regardless of the tax-compliance style. Theta oscillations are one of the dominant rhythms in the frontal cortices and are considered the fingerprint of all limbic structures^34^, which constitute the core network for affective experience and responses. Frontal theta activity, in particular, is thought to mirror emotional reactions, affectively-connoted arousal, and emotionally-loaded states following the emotional appraisal process^35–39^. While the SCL modulation by itself might have been read as a consequence of increased cognitive workload due to the complexity of the virtual social context, the peculiar functional associations of theta activity depose in favour of the affective connotation of the effect exerted by the social frame, as opposed to an alternative purely cognitive explanation. The affective connotation of the social frame may relate to an increased sense of social belonging and the subsequent social norms that are elicited (i.e., reciprocity).

Such interpretation is supported even by findings concerning the psycho-physiological correlation between behavioural and autonomic data – namely, HR values – specifically when participants made their choices in the social condition. Whereas the systematic preference of the whole sample for compliant choices and the lack of significant difference between experimental conditions (individual vs. social) when focusing only on behavioural data (i.e. participants decision to pay taxes or not during the experimental task) may be plausibly justified by the implicit influence of social desirability and observer effect on overt behaviour, such across-levels analysis shows the potential of integrating measures able to tackle both overt and covert correlates of explicit behaviour and related cognitive processes.

The fact that lower rates of compliant responses (i.e. decisions to pay taxes) was associated to increased HR values only in the social condition corroborates the hypothesis that even just a social frame can influence implicit attitudes and covert physiological activation when waiting for a potential feedback following socially-inappropriate or unethical decisions.

While such across-level association may open interesting windows on the relationship between overt behaviour and latent psychosocial factors influencing the decision-making process, we acknowledge that such issue is worth further investigation. In fact, despite the consistent pattern of physiological activation presented by participants in the social condition, no significant differences were found on behavioural data between experimental conditions. Bigger samples and replication studies may properly strengthen theoretical interpretation and practical implications, also allowing to delve deeper into the potential mediation role of individual forms of tax-compliance. Besides being affected by the sample size, the lack of significant differences at behavioural level may also be explained by the relatively weak manipulation between experimental conditions. The social condition was only different in that participants were reminded of the presence of other participants, but the individual and the social conditions did not vary in terms of consequences of tax compliance choices. Still, this kind of manipulation – although apparently not strong enough to affect behaviour – was successful in influencing covert physiological activation and latent processes, which was the main aim of this study.

A second main result concerns a critical comparison between people presenting a voluntary or an enforced tax-compliance style. Namely, values of heart rate were higher when, during the feedback phases, participants were not presented with a fiscal audit than when they received an audit, and such difference specifically characterized the enforced tax-compliance group. The increase of heart rate is typically considered a marker of heightened physiological activation, should it depend from a transient orienting response – i.e. an automatic increase of arousal and implicit orientation of attention to relevant environmental modification and salient stimuli – or a more stable arousing condition^30,40^. Given the structure of the task and the fact that the HR increase was recorded when the fiscal audit was not presented to participants, we suggest that the observed rise of physiological arousal was influenced by expectations and mirrored a form of pleasant activation due to the lack of the examination. In line with such interpretation, the group showing such autonomic response was composed by people with higher levels of enforced tax-compliance – i.e. a propensity toward tax-payment that primarily grounds on taxpayers’ concerns of being audited and fined. Since these people are actually driven to pay taxes by the possibility of being punished in case they would be found non-compliant, being notified that they would not be asked to undergo a fiscal examination plausibly elicited a positive pleasant feeling and a related activation response. The absence of further relevant task-induced differences in electrodermal and electroencephalographic activity, however, prevents us from outlining a better-defined scenario.

In addition, no significant differences between tax compliance styles (enforced vs. voluntary) were found on behavioural data. This result, however, is not surprising and is in line with the ‘slippery slope’ framework. Both voluntary and enforced tax compliance styles may lead to high levels of tax compliance: the former because of taxpayers’ moral obligation towards the common good, the latter because of taxpayers’ fear of audits and sanctions. In this study, participants were aware of possible audits throughout the whole experiment, thus even participants with an enforced tax style were compelled to be compliant. Again, this study shows how similar overt behavioural responses may actually derive by different motives and latent processes.

To our best knowledge, the present experiment is one of the few studies constituting a novel and promising branch of neuroeconomics research, i.e. the application of neurosciences for the investigation of tax-compliance. We support the idea that classic economic theories of tax behaviour developed under the assumption that taxpayers act as rational and individualist agents do not provide a realistic and comprehensive account for the decision-making process. Other social and psychological variables play a significant role, as suggested by the ‘slippery slope’ framework^13^. Reported findings add to available evidence highlighting the contribution of psychological and social-affective variables to individuals’ decision-making processes to pay or evade taxes and to their appraisal of the consequences of such choice. Thus, the importance of combining the economic and psychological perspectives in tax behaviour research is also supported at neurobiological level.

Nonetheless, the study presents different limitations. Firstly, the structure of the task we used is different from traditional social economic games, such as the ultimatum game, where agents’ choices and feedbacks are explicit and can consequently directly modulate the development of their economic decisions. We opted for the reported solution because we wanted to recreate the peculiar situation of tax-payment, which is connoted as an individual independent decision that nonetheless has shared consequences on social life and public welfare. Still, such choice made comparison with literature on the topic, which is yet quite scarce, even more complex. Future investigation might benefit from pairing traditional and *ad hoc* economic games so to support data interpretation and broaden conclusive remarks. Secondly, the pattern of significant autonomic modulations observed in the social and in the non-audit conditions, as well as in association to behavioural data, was not complete. Additional investigations taking into account even other measures of autonomic activity (e.g. heart rate variability) are worth in order to sketch a clearer view of latent variables and of somatic markers influencing decision-making concerning tax-payment and the evaluation of the consequences of that choice.

## Data Availability

The datasets generated during and/or analysed during the current study are available from the corresponding author on reasonable request.
